# Were Our Grandmothers Right? Soup as Medicine—A Systematic Review of Preliminary Evidence for Managing Acute Respiratory Tract Infections

**DOI:** 10.3390/nu17132247

**Published:** 2025-07-07

**Authors:** Sandra Lucas, Matthew J. Leach, Rachel Kimble, Joshua Cheyne

**Affiliations:** 1 School of Health and Life Sciences, University of the West of Scotland, Lanarkshire G72 0LH, UK; rachel.kimble@uws.ac.uk; 2 Joanna Briggs Institute (JBI), University of Adelaide, Adelaide 5005, Australia; matthew.leach@adelaide.edu.au; 3 Library, University of the West of Scotland, Paisley PA1 2BE, UK; joshua.cheyne@uws.ac.uk

**Keywords:** complementary medicine, food, nutrition, respiratory infection, soup, systematic review, traditional medicine, food as medicine, nutritional intervention

## Abstract

Background/Objectives: Acute respiratory tract infections (ARTIs) are a significant global health burden, contributing to increased healthcare use, absenteeism, and economic strain. While clinical treatments exist, many individuals use traditional dietary remedies such as soup to relieve symptoms. Soup is thought to support recovery through hydration, warmth, nutritional content, and possible anti-inflammatory effects. This systematic review aimed to evaluate the therapeutic effects of soup consumption on adults with ARTIs, focusing on symptom severity, illness duration, absenteeism, immune response, inflammatory biomarkers, and overall well-being. Methods: A systematic literature search was conducted in February 2024 across MEDLINE, Scopus, CINAHL, the Cochrane Library, clinical trial registries, and supplementary sources. Eligible studies included randomized controlled trials, non-randomized trials, and controlled before-after studies evaluating soup as an intervention for ARTIs. Two reviewers independently screened studies, extracted data, and assessed risk of bias using the Cochrane Risk of Bias 2 tool. A narrative synthesis was undertaken due to heterogeneity in study design and outcome measures. The protocol was registered with PROSPERO (CRD42023481236). Results: Four studies (n = 342) met inclusion criteria. Interventions commonly included chicken-based soups with vegetables and herbs. Comparators varied (e.g., no treatment, water, or alternative soup). Findings showed modest reductions in symptom severity and illness duration (by 1–2.5 days). Two studies reported reductions in inflammatory biomarkers (IL-6, TNF-α, CRP). No studies reported on absenteeism or well-being. Conclusions: Soup may offer modest benefits for ARTIs, particularly for symptom relief and inflammation. Further well-designed studies are needed to evaluate its broader clinical and functional impacts.

## 1. Introduction

Acute respiratory tract infections (ARTIs) are a significant global health concern, contributing substantially to illness, healthcare costs, and mortality. In 2019, upper respiratory infections accounted for 17.2 billion incident cases, representing 42.8% of all causes reported in the Global Burden of Disease study GBD 2019 Diseases and Injuries Collaborators [1]. Vulnerable populations, particularly the elderly and children aged under five years, face the highest risk of severe outcomes, including fatalities [2]. For adults and caregivers of children with ARTIs, the impact often extends beyond physical symptoms to include social and economic burdens such as work absenteeism, reduced productivity, and stress [3].

Current management strategies for ARTIs predominantly rely on pharmaceutical treatments such as antipyretics, analgesics, and decongestants. While these interventions are widely used, their effectiveness is sometimes limited, and concerns about potential side effects, particularly in children, have led to increased interest in alternative approaches [4]. Traditional, Complementary and Alternative Medicine (TCAM) practices have gained traction as adjunctive or standalone therapies for ARTI. Among TCAM interventions, dietary therapies such as soup are particularly popular due to their accessibility, cultural acceptability, and perceived safety [5,6].

Soup has long been used as a home remedy for respiratory illnesses, cherished as a “grandmother’s cure” and steeped in cultural tradition. Often recommended by TCAM practitioners and embraced by families worldwide, it holds a unique place in both folklore and integrative healthcare. Soup is considered a “food as medicine” intervention, believed to provide hydration, essential nutrients, and anti-inflammatory properties that may alleviate symptoms and support immune function [7]. Existing research indicates that soup consumption is associated with modest improvements in symptom severity, shortened illness duration, and potential enhancements in overall well-being [4,8]. However, despite its widespread use and generational endorsement, scientific evidence validating these claims remains limited.

This systematic review aims to address this evidence gap by evaluating the potential benefits of soup in managing ARTIs in adults. By consolidating current evidence, the review seeks to clarify soup’s therapeutic role and its potential integration into contemporary respiratory care. The findings are intended to inform clinical practice and inspire future investigations into the mechanisms and effectiveness of dietary interventions for ARTI management.

## 2. Methods

### 2.1. Study Design

**Figure 1:**
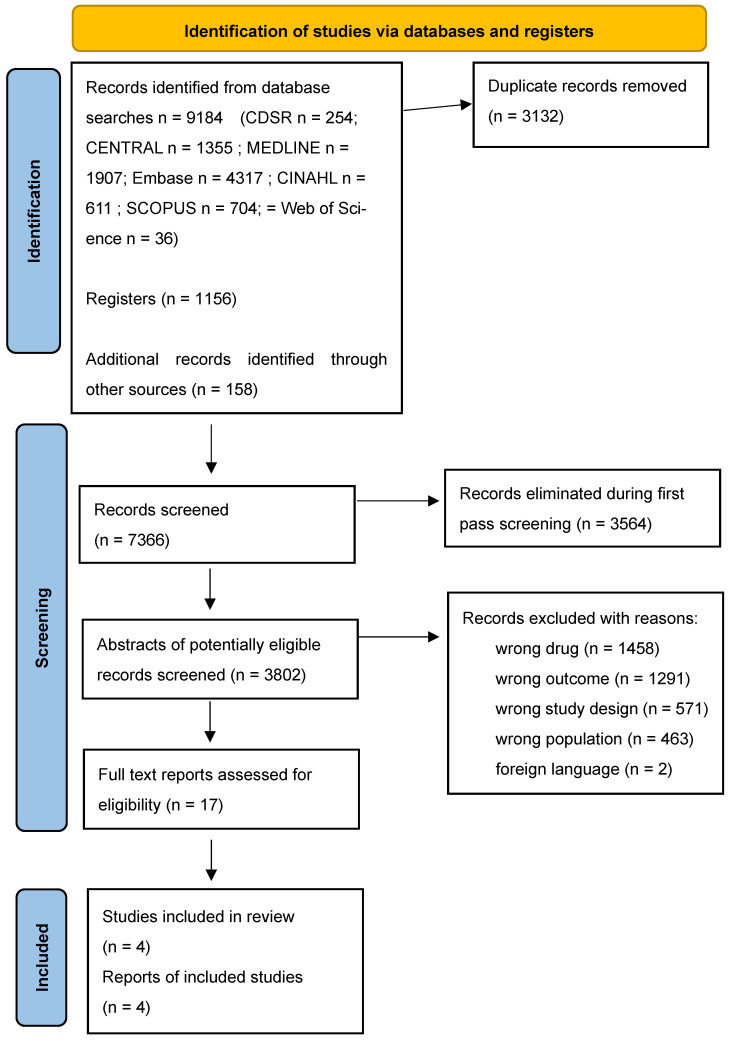
PRISMA 2020 flow diagram.

This review employed a JBI-aligned systematic review of effectiveness approach [9], registered with PROSPERO and reported in accordance with Preferred Reporting Items for Systematic Reviews and Meta-Analysis (PRISMA) framework (Figure 1) to ensure transparency, rigour, and reproducibility [10,11,12]. Prior to conducting this systematic review, a scoping review was undertaken to assess the breadth of available literature. This preliminary review screened 1262 articles, identifying two studies that met the inclusion criteria for further in-depth analysis. The findings from the scoping review provided a foundation for refining the research question, inclusion criteria, and search strategy of the systematic review.

### 2.2. Objectives

The objective of this systematic review was to synthesise the evidence regarding the effects of soup on adults with ARTI. Specifically, the review explored the effectiveness of soup in managing ARTI, focusing on its impact on symptom severity, illness duration, absenteeism, immune response and inflammatory biomarkers as well as overall well-being. We defined soup as a savoury liquid meal, comprising vegetables, meats, cereals and/or fruits, and water or stock as a base [13].

### 2.3. Inclusion and Exclusion Criteria

The systematic review search strategy for the MEDLINE Ovid database was developed in consultation with an Academic Librarian to ensure both comprehensiveness and precision (see Appendix A). The search was guided by a Participant-Intervention-Comparator-Outcome-Study design (PICOS) framework (Table 1) to define the inclusion and exclusion criteria. Studies were eligible if they involved participants with acute respiratory tract infections (ARTIs) and evaluated soup as a therapeutic intervention. While the review was primarily focused on adults, studies involving children were not excluded if they met all other inclusion criteria.

### 2.4. Data Sources

Searches were conducted on 6th February 2024 using the following databases: MEDLINE Ovid (from 1946) (Appendix A); Cochrane Central Register of Controlled Trials (CENTRAL) and the Cochrane Database of Systematic Reviews (CDSR) in The Cochrane Library; Informit Health (from 1945); CINAHL EBSCO (Cumulative Index to Nursing and Allied Health Literature; from 1937); and Scopus (2004).

### 2.5. Search Strategy

The search strategy employed a mix of Medical Subject Headings (MeSH) and uncontrolled vocabulary/keywords to locate relevant literature. The search terms were clustered into three categories: ARTI, intervention (i.e., soup or broth as a therapeutic intervention), and study design. For the latter, we used the Cochrane Highly Sensitive Search Strategy for identifying randomised trials in MEDLINE Ovid: sensitivity-maximising version (2008 revision), as described in the Cochrane Handbook for Systematic Reviews of Interventions, 4.S1 Technical Supplement to Chapter 4: Searching for and selecting studies (lines 34–41) [12]. This search strategy served as the foundation for all database searches included in this review.

Additionally, supplementary searches included grey literature repositories (e.g., Trove, Grey Literature Report), the US National Institutes of Health Ongoing Trials Register ClinicalTrials.gov (clinicaltrials.gov/), and World Health Organisation International Clinical Trials Registry Platform (ICTRP; trialsearch.who.int). Boolean operators and truncation were applied to combine search terms effectively. Duplicate records were removed using EndNote 21™ (Clarivate, Philadelphia, PA, USA) software.

### 2.6. Study Identification and Selection

All records identified through database searches were uploaded into Rayyan© for systematic screening. Screening was conducted in two phases:

1. Title and Abstract Screening: Articles were assessed for relevance against the predefined inclusion and exclusion criteria by two independent reviews.
2. Full-Text Screening: Eligible full-text articles were retrieved and reviewed independently by two reviewers. Discrepancies were resolved through discussion or a third reviewer.

### 2.7. Data Extraction

A standardised data extraction form was used to collect the following information:

Study ID, author(s), and publication year;

Study location and setting;

Participant demographics (e.g., age, gender, ARTI diagnosis);

Intervention details (e.g., type, content, and duration of soup treatment);

Outcomes measured (e.g., symptom severity, illness duration, absenteeism, immune response, well-being); and

Study design and sample size.

### 2.8. Assessment of Publication Bias

The methodological quality of the included studies was appraised using the National Health and Medical Research Council (NHMRC) levels of evidence [14], in alignment with JBI guidance, which classifies evidence based on study design. Risk of bias was further assessed using the Cochrane Risk of Bias 2 (RoB 2) tool for randomised controlled trials, which evaluates five domains to generate an overall judgement of bias as low, high, or raising some concerns [15]. Assessments were conducted independently by two reviewers, with disagreements resolved by a third reviewer.

### 2.9. Outcomes

The primary outcome of the review was well-being, which was defined as a state of feeling good and functioning well [16]. Well-being could be measured using any well-being and/or health-related quality of life outcome measure. Secondary outcomes included: symptom severity (i.e., the intensity of ARTI-related symptoms), illness duration (i.e., the length of time from ARTI onset to the cessation of ARTI symptoms), absenteeism (i.e., the period of absence from school or work due to ARTI) and immune response and inflammatory biomarkers (i.e., *C*-reactive protein; immunoglobin; interleukin-1 beta; interleukin-6; interleukin-17; interleukin-10; quantitative *C*-reactive protein; tumour necrosis factor-alpha; and interferon-gamma). Secondary outcomes could be assessed using any outcome measure.

### 2.10. Data Synthesis

Data were synthesised narratively due to clinical and methodological heterogeneity across the included studies. This heterogeneity arose from variations in soup formulations, participant characteristics, intervention protocols, and outcome measures. The synthesis process comprised the following steps:

Descriptive Summaries: Characteristics and findings of included studies were summarised and tabulated, including study design and intervention components.

Outcome Categorisation: Results were grouped by primary outcome domains—well-being, symptom severity, illness duration, absenteeism, and immune response.

Subgroup Analysis: Narrative comparisons were conducted to explore outcome differences based on soup type, intervention duration, and study setting.

Limitations: Methodological limitations and gaps in the evidence base were identified and described.

Meta-analysis was not conducted due to the small number of eligible studies and the substantial variability in interventions and reported outcomes. Future updates to this review may incorporate pooled analyses as more data become available.

The certainty of evidence was not formally assessed using GRADE, owing to the limited number and heterogeneity of the included studies.

## 3. Results

### 3.1. Overview of Search Results

The searches identified a total of 10,598 records (see PRISMA Figure 1). After removing 3134 duplicates, 7349 records were screened. Of these, 3564 titles were excluded in the first pass, leaving 3802 abstracts for further review. Following abstract screening, 3785 abstracts were eliminated as irrelevant, reducing the pool to 17 full-text studies for evaluation. Four studies met the inclusion criteria. Reasons for exclusion included irrelevant outcomes (3749), inappropriate study design (1072), and wrong population (928).

### 3.2. Characteristics of Included Studies

Of the final four included studies comprised four randomised controlled trials (RCTs). The studies were conducted across three broad regions, including North America (*n* = 1), and Asia (*n* = 3). The pooled sample size across the five studies was 342 participants, with sample sizes ranging from 15 to 160. The samples included children, adults, and older adults diagnosed with ARTIs (such as the common cold, influenza-like illnesses, and pharyngitis).

The included studies used diverse interventions and outcome measures. Interventions primarily consisted of chicken-based soups enriched with vegetables, with control groups receiving either no intervention or alternative beverages (e.g., water or tea). Comparators varied across studies and included barley soup, hot or cold water, usual care, or alternative beverages. In one case, a pre-post comparison within a single group was used [17]. Unlike the other included studies, Wang, Zhang [17] employed a pre-post design within a single cohort, without an external comparator group.

Primary outcomes included symptom severity, duration of illness, well-being, absenteeism from school or work, and immune and inflammatory biomarkers. Further details of the included studies are summarised in Table 2.

### 3.3. Description of Interventions

The soups evaluated in the four included studies incorporated a diverse range of ingredients (Table 3). Study 1 [18] featured a complex blend of grains (peeled wheat, rice, mung), legumes (pea, cowpea), vegetables (carrot, onion, spinach, beets), and a variety of herbs and spices (parsley, coriander, mint, pennyroyal, celery seeds), emphasising both anti-inflammatory and antioxidant benefits. Study 2 [19] utilised commercially available chicken soup, serving as a simple and practical intervention. Study 3 [20] combined traditional plant-based ingredients like *Ficus carica*, *Vitis vinifera*, and safflower with chicken and barley soups, further enriched with rose water, saffron, and cinnamon for their immune-supportive and aromatic properties. Lastly, Study 4 [17] featured a medicinal herbal soup based on traditional Chinese medicine, incorporating ginseng, ginger, cinnamon bark, and other roots known for their immune-modulating and anti-inflammatory effects.

### 3.4. Assessment of Risk of Bias

**Figure 2:**
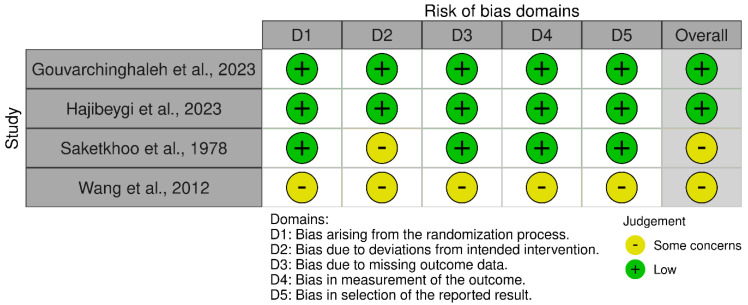
Risk of bias for included studies, dot plot [17,18,19,20].

Random sequence generation was adequately described in three of the four included studies, although two studies lacked detailed procedures for allocation concealment. Due to the nature of dietary interventions, blinding was inherently challenging. Accordingly, participants were often aware of their treatment group, increasing the risk of performance bias. Detection bias was less significant as outcomes were primarily self-reported or objective, such as biomarkers. Attrition rates were low (<10%) across all studies, reducing the risk of bias from missing data. In particular, there were some concerns in the reporting in 2 of the studies [17,19]. The overall risk of bias scores for each study is summarised in Figure 2.

### 3.5. Effects of Interventions

Although well-being and absenteeism were designated as primary outcomes in this review, none of the included studies reported data on these measures. This omission underscores a critical evidence gap in evaluating holistic recovery outcomes such as absenteeism and perceived well-being. This represents a critical evidence gap, particularly given the widespread socioeconomic impact of ARTIs and the potential for soup to influence recovery-related absenteeism and perceived wellness.

#### 3.5.1. Duration of ARTI

One study [20] examined changes in ARTI duration. The study [20] demonstrated a statistically significant reduction in illness symptom duration, with the intervention group recovering earlier than control. The remaining studies [17,18,19] did not report duration of illness or symptoms.

#### 3.5.2. Severity of ARTI Symptoms

Three studies tested severity of ARTI symptoms, with all studies reporting significant reductions in ARTI symptom severity, particularly nasal congestion, sore throat, and cough, with soup consumption. Study 2 [17] identified the change in nasal mucus velocity. Study 3 [20] reported significant reductions in pro-inflammatory cytokines following consumption of chicken and vegetable broth, which correlated with ARTI symptom relief. Study 4 [17] found consumption of chicken soup in children improved cough and sore throat severity.

#### 3.5.3. Immune Response and Inflammatory Biomarkers

Two studies [17,18] evaluated immune and inflammatory responses to soup interventions. Study [17] demonstrated significant reductions in pro-inflammatory cytokines (IL-6 and TNF-α) and reported enhanced lymphocyte proliferation and activation in participants consuming chicken-based soup, indicating improved systemic immune readiness. Study [18] found that soup significantly improved inflammatory markers (IL-1β, IL-6, IL-17, IL-10, TNF-α) and metabolic indicators (D-dimer, blood urea nitrogen, and creatinine); however, the control group more effectively reduced CRP and potassium levels (*p* < 0.05).

## 4. Discussion

This systematic review evaluated the role of soup as a complementary therapy for the management of acute respiratory tract infection in adults. The review explored the effects of soup on key outcomes, including symptom severity, duration of illness, absenteeism, and overall well-being. Findings indicate that soup consumption was associated with modest reductions in symptom severity, suggesting potential therapeutic benefits for individuals with ARTIs through its combined effects of hydration, nutritional support, and anti-inflammatory properties. The discussion that follows contextualises these findings within existing research, examining their practical and clinical implications, and highlighting opportunities for future investigations of soup as an adjunctive intervention for the management of ARTI.

### 4.1. Soup as a Multifaceted Remedy

The findings of this review highlight soup’s potential to alleviate ARTI symptoms. While the mechanisms of action of soup are not entirely clear, evidence suggests that soup, particularly chicken soup, may demonstrate anti-inflammatory effects, possibly due to its protein content [21]. This aligns with complementary dietary approaches such as Mediterranean and plant-based diets, which have been shown to enhance the immune response through nutrient synergy [3,8,22].

The addition of vegetables further augments the benefits of chicken soup by increasing its antioxidant and anti-inflammatory properties [5,22,23]. This is consistent with historical and cultural observations of soup’s healing properties, as noted in its use since Greco-Roman times for respiratory relief [22,24]. By combining hydration, nutritional value, and thermal effects, soup has been associated with a broader range of therapeutic outcomes than standalone remedies such as herbal teas [4,25].

Although the included studies varied in their soup formulations, several common components such as chicken protein [7], root vegetables (e.g., beets, onions) [26,27,28], and traditional herbs have known anti-inflammatory, antioxidant, or immune-supportive properties. Chicken protein may contribute to mucosal repair and immune modulation, while vegetables provide micronutrients and phytochemicals that support immune defence [29]. Herbs such as ginger [30] and garlic [31,32], present in some preparations, are also associated with respiratory and immune benefits in traditional and nutritional medicine literature

### 4.2. Impact on Recovery Duration

The modest reductions in illness duration observed in this review are consistent with the findings of other studies. Indeed, dietary studies, such as those conducted by van der Gaag, Brandsema [22], have revealed similar benefits, with nutrient-rich diets significantly reducing ARTI duration and severity decreasing antibiotic use [3,22,31]. Further, the combined effects of hydration and immune-supportive nutrients in soups are analogous to the benefits observed with micronutrient supplementation, which similarly have been shown to modulate inflammatory responses, as well as strengthen epithelial integrity [8,22]. These findings highlight soup’s potential as a holistic, food-based intervention in managing ARTI-related morbidity. By reducing illness burden and work/school absenteeism, the consumption of soup for ARTI therefore has significant public health and economic implications.

### 4.3. Hydration and Symptom Relief

Hydration may be an integral mechanism through which soup alleviates ARTI symptoms. Warm fluids, including soup, stimulate mucous clearance and enhance nasal airflow, thereby offering immediate relief from nasal congestion. This hypothesis has been corroborated by studies comparing hot soup with other warm liquids like teas and broths [23].

The dual benefits of hydration and nourishment distinguish soup from other fluid-based interventions. For instance, the high nutrient density in soup, including phytochemicals [32], zinc, and vitamins C and A, contributes to epithelial repair and immune support, as seen in studies on micronutrient-enriched diets [8,31]. Notwithstanding, without further investigation, it is not possible to determine which, if any of these components, remain bioactive after cooking, and therefore play a crucial role in the therapeutic activity of soup.

### 4.4. Cultural Acceptance and Accessibility

The widespread cultural familiarity and low cost of soup make it uniquely suited for integration into ARTI management plans. Unlike many TCAM interventions, soup enjoys universal acceptance across populations and regions [3,24]. This makes soup particularly valuable in resource-limited settings, where it can serve as a cost-effective intervention for managing respiratory conditions [25]. This potentially positions soup as a practical tool for public health, particularly in resource-constrained settings where access to conventional therapies may be limited. Nevertheless, economic evaluations of soup for ARTI are currently lacking, and are necessary to determine the cost–benefit of this intervention in resource-constrained settings.

The tailored development of region-specific soups, such as vegetable-enriched chicken soups in Vietnam, underscores its versatility and adaptability as a dietary intervention [3,5]. These findings align with evidence on functional foods like plant-based protein diets, which have been found to reduce respiratory illness rates and improve overall health outcomes [8,31].

### 4.5. The Role of Soup in Integrated Healthcare

The findings of this review align with the broader concept of “food as medicine,” emphasising the importance of integrating culturally relevant, low-cost, and evidence-informed dietary interventions into public health strategies. The GRADE Evidence to Decision framework underlines the significance of acceptability, equity, and feasibility in health system and public health decisions [33]. These principles reinforce soup’s potential as a low-risk, accessible adjunct to conventional ARTI treatments, bridging the gap between clinical nutrition and holistic care approaches.

In summary this review is the first to systematically evaluate the effects of soup as an intervention for ARTIs, highlighting its potential as a culturally embedded, low-risk therapeutic option. Although preliminary, the evidence indicates that soup consumption is associated with modest reductions in symptom severity and modulation of inflammatory biomarkers. Given its accessibility and acceptability, soup shows potential as an adjunctive dietary intervention in clinical and public health settings.

### 4.6. Strengths and Limitations of the Systematic Review

This systematic review demonstrates several key strengths, establishing its credibility and significance in an emerging area of research. To our knowledge, this is the first systematic review to evaluate the effects of soup on acute respiratory tract infections (ARTIs), addressing a novel intervention with broad implications for contemporary healthcare. Methodologically, the review adhered to PRISMA guidelines, and the protocol was prospectively registered, ensuring transparency and minimising reviewer bias. Additionally, the comprehensive search strategy developed and conducted by an experienced information specialist utilised multiple databases and included grey literature, maximising the likelihood of identifying all relevant studies. These strengths provide a solid foundation for the review’s findings and its contribution to the emerging evidence base on culturally embedded, food-based health interventions.

This review also faced several limitations, though each presents an opportunity for future improvement. The small number of included studies limits generalisability but highlights the novelty of the topic. Notably, although well-being and absenteeism were predefined as primary outcomes, none of the included studies assessed these measures. This omission represents a critical gap in the evidence and underscores the need for future studies to include broader indicators of recovery and functional impact.

The geographic concentration of studies, three of the four conducted in Asia, may restrict the applicability of findings across diverse cultural and dietary contexts. This geographic imbalance highlights the need for further research across diverse global populations. One included study involved a paediatric population; it was retained due to its relevance to the intervention and its contribution to understanding immune-related outcomes. Heterogeneity in soup formulations, intervention durations, and outcome measures complicates synthesis but also reflects the intervention’s cultural flexibility and global relevance. While self-reported outcomes are prone to bias, they offer valuable patient-centred insights and could be supplemented with objective biomarkers in future research. Blinding remains a methodological challenge in dietary interventions; however, the use of standardised control comparators, such as alternative warm beverages, may help minimise bias.

Although GRADE was not formally applied due to the small number and heterogeneity of studies, a narrative estimate suggests that the overall certainty of evidence is low to moderate. This judgement is based on methodological quality, sample sizes, outcome consistency, and risk of bias across the included studies. This transparent assessment underscores the preliminary nature of the findings and the need for high-quality, standardised research to strengthen confidence in soup as an adjunctive intervention for ARTIs. While the observed benefits are biologically plausible, caution is warranted when interpreting these early findings.

### 4.7. Future Directions

The tradition of using chicken soup to alleviate cold symptoms, often passed down from generations, underscores its enduring place in home remedies and cultural practices. Despite its popularity, scientific validation of its therapeutic effects remains limited, highlighting the need for further investigation. Future research should prioritise high-quality, large-scale randomised controlled trials to evaluate the efficacy of soup as an adjunctive therapy for ARTIs. Standardising soup recipes and intervention protocols across studies would enhance comparability and clarify the specific active components and mechanisms of action.

To provide a more comprehensive understanding of the efficacy of soup for ARTI, future studies should consider both objective clinical measures, such as inflammatory biomarkers and symptom severity scales, and patient-centred outcomes, such as comfort and quality of life. Expanding research to diverse geographic and cultural contexts would not only validate findings globally, but also adapt interventions to regional dietary practices, ensuring cultural relevance and evidence implementation. Additionally, assessing the cost-effectiveness of soup interventions, particularly in resource-limited settings, could strengthen their role in public health strategies. Exploring the integration of soup-based therapies within conventional healthcare systems, supported by practitioner and patient education initiatives, would further promote its adoption as an accessible and culturally resonant therapy. These research directions will bridge the gap between tradition and evidence, paving the way for integrating soup as an effective adjunct to ARTI management.

## 5. Conclusions

This systematic review highlights the potential role of soup in the management of ARTIs, offering benefits in reducing symptom severity, promoting hydration, and supporting overall well-being. The findings underscore the potential therapeutic value of combining nutrition, hydration, and anti-inflammatory properties, which make soup a biologically plausible, culturally relevant and accessible intervention. Despite the promising results reported in this review, limitations such as study heterogeneity and a lack of standardised protocols highlight the need for more rigorous and diverse research to validate these findings. In addressing these evidence and knowledge gaps through future research, we will better determine whether soup is an effective, practical adjunct to conventional ARTI care, for both individuals and healthcare systems alike. This review lays the groundwork for further exploration and underscores soup’s potential as a low-risk, culturally embedded intervention worthy of more rigorous clinical evaluation.

Given the variability and limitations of the included studies, and in the absence of a formal GRADE assessment, we estimate the certainty of the current evidence to be low to moderate. This provides a foundation for future high-quality trials to clarify the role of soup as a dietary intervention in respiratory care.

## Figures and Tables

**Table 1:** Study inclusion and exclusion criteria.

Concept	Inclusion Criteria	Exclusion Criteria
**Population**	Studies involving adults with ARTI (e.g., upper respiratory tract infection, common cold, influenza, COVID-19)	Studies on chronic respiratory conditions (e.g., asthma, cystic fibrosis)
**Intervention**	Soup or broth as a therapeutic intervention	Studies examining soup or broth in combination with other interventions, where the effects of soup cannot be isolated.
**Comparator**	No intervention, placebo, alternative beverage, complementary medicine or pharmaceutical medicine	None
**Outcomes**	Duration of symptoms, severity of symptoms, absenteeism and/or well-being, immune response and inflammatory markers	None
**Study design**	Controlled before-after studies, controlled clinical trials, non-randomised controlled trials and randomised controlled trials	Qualitative research, systematic reviews, case studies, editorials, in vitro, and comparative studies

**Table 2:** Characteristics of included studies (*n* = 4).

Study	Study ID	Design	Population	Intervention	Comparator	Outcomes	Results	RoB
1 [18]	Gouvarchinghaleh et al. (2023)	RCT	Adults (*n* = 60)	Functional food soup (7 days)	Barley soup	Inflammatory cytokines (IL-1β, IL-6, IL-17, IL-10, CRP, TNF-α, IFN-γ)	Significant reductions in IL-1β and IL-6 in intervention group. Greater improvement in intervention group in terms of IL-1β; 74.0 ± 12.5 vs. 63.8 ± 11.7 pg/mL (*p* = 0.002) and IL-6; 54.55 ± 9.81 37.6 ± 6.8 pg/mL (*p* < 0.001).	Some concerns
2 [19]	Saketkhoo et al. (1978)	RCT	Adults (*n* = 15)	Chicken + vegetable soup (single administration)	Sham drink (cold water or hot water)	Nasal mucus velocity and airflow	Increased nasal mucus velocity post-intervention compared to sham. Five minutes after drinking hot chicken soup by sip, mean nasal mucus velocity rose by 2.3 mm per minute over baseline, after drinking hot chicken soup by straw by 1.4 mm per minute, both significantly different from sham.	High
3 [20]	Hajibeygi et al. (2022)	RCT	Adults (*n* = 160)	Chicken + barley soup with traditional herbs (5 days)	Usual care or alternative beverage	Symptom severity, CRP, WBC, fatigue, hospitalisation	Reduced symptom severity and CRP levels; shorter illness duration. In the intervention group mean difference for weakness 0.8 (95% CI: −0.14–1.74; *p* = 0.031), and cough 0.44 (95% CI: 0.09–0.78; *p* = 0.043). Lower CRP, mean difference 2.26 pg/mL (*p* = 0.045). Length of symptoms shortened cough 6.19 ± 1.31–6.31 ± 1.97 (*p* = 0.118 sputum 1.88 ± 2.13–2.2 ± 2. 35 (*p* = 0.445) headache 1.55 ± 2.07–2.35 ± 2.72 (*p* = 0.190) myalgia 6.23 ± 1.26–6.75 ± 1.44 (*p* = 0.086) GI 1.15 ± 1.97–2 ± 3.17 (*p* = 0.461) and length of hospitalisation shortened 6.55 ± 1.17–6.95 ± 1.33 (*p* = 0.097)	Low
4 [17]	Wang et al. (2012)	RCT	Children (*n* = 107)	Double Fill Nine Tastes Soup (2 months)	Before-after comparison within the same cohort (no external comparator group)	Recurrent infections, immunoglobulins, cytokines	Reduced infection frequency; increased Ig levels; lower TNF-α. The intervention group experienced fewer recurrent infections per year (≥14 before) reduced to ≤5 in 20 cases. After two months, the intervention group had notably higher levels of immunoglobulins (IgA: 0.84 vs. 0.53 g/L, IgG: 8.41 vs. 4.59 g/L, IgM: 1.17 vs. 0.74 g/L) and cytokines (IL-12: 48.16 vs. 23.19 ng/L, INF-γ: 56.27 vs. 40.45 ng/L), and lower TNF-α levels (40.06 vs. 72.21 ng/L), indicating improved immune function.	Some concerns

Note: *C*-reactive protein (CRP); immunoglobulin (Ig); interferon-gamma (IFN-γ); interleukin-1 beta (IL-1β); interleukin-6 (IL-6); interleukin-10 (IL-10); interleukin-17 (IL-17); randomised controlled trial (RCT); tumour necrosis factor-alpha (TNF-α).

**Table 3:** Soup ingredients reported in each study.

Study Number	Intervention Duration	Ingredients
Study 1 [18] Gouvarchinghaleh, H.E.; et al. (2023)	7 days	Peeled wheat, rice, mung, pea, apple, quince, carrot, cowpea, almond, onion, fresh garlic, parsley, coriander, leeks, mint, pennyroyal, spinach, beets, ground celery seeds, alfalfa sweat, yarrow sweat, chamomile sweat, olive oil, roasted sesame, roasted black seed, and roasted turmeric. Control: Barley soup.
Study 2 [19] Saketkhoo, K.; et al. (1978)	Single administration	Chicken soup from market.
Study 3 [20] Hajibeygi R.; et al. (2022)	5 days	*Ficus carica*, *Vitis vinifera*, *Cicer arietinum*, *Descurainia sophia* seeds, safflower, *Ziziphus jujuba*, chicken soup; barley soup, rose water, saffron, and cinnamon spices.
Study 4 [17] Wang, Y.; et al. (2012)	2 months	Ginseng, Astragalus root, *Angelica sinensis*, *Poria cocos*, liquorice root, peony root, cinnamon bark, ginger, jujube fruit.

## Data Availability

All data generated or analysed during this systematic review are included in this published article or referenced in publicly available databases.

## Abbreviations

ARTI	Acute respiratory infection
COVID-19	Coronavirus Disease 2019
TCAM	Traditional Complementary and Alternative Medicine
URTI	Upper Respiratory Tract Infection

## Appendix A. Search Strategies

**The Cochrane Library (CDSR and CENTRAL) search strategy**

#1 MeSH descriptor: [Respiratory Tract Infections] this term only
#2 MeSH descriptor: [Nasopharyngitis] this term only
#3 MeSH descriptor: [Sinusitis] explode all trees
#4 MeSH descriptor: [Rhinitis] this term only
#5 MeSH descriptor: [Sneezing] this term only
#6 MeSH descriptor: [Pharyngitis] this term only
#7 MeSH descriptor: [Tonsillitis] this term only
#8 MeSH descriptor: [Laryngitis] explode all trees
#9 MeSH descriptor: [Tracheitis] this term only
#10 MeSH descriptor: [Epiglottitis] this term only
#11 (acute near/3 (chest or airway* or bronchopulmonary or pulmonary or respirat*) near/3 disease*):ti,ab,kw
#12 ((respirat* near/2 (failur* or distress* or infection* or inflamm* or disease* or congest* or syndrome*)) or (pain* near/2 (respir* or breath*))):ti,ab,kw
#13 (URTI or URI or LRTI or LRI or ARI or ARTI):ti,ab,kw
#14 (nasopharyngit* or rhinopharyngit* or sinusit* or rhinit* or rhinosinusit* or nasosinusit*):ti,ab,kw
#15 (((runny or running or blocked or congest* or discharg* or stuffed or stuffy) near/2 (nose* or nasal)) or rhinorrhea or rhinorrhoea or sneez* or pharyngit* or pharyngotonsillit*):ti,ab,kw
#16 (throat* near/2 (sore or infect* or inflam*)):ti,ab,kw
#17 (tonsillit* or laryngit* or croup* or laryngotracheobronchit* or pseudocroup* or tracheit* or rhinotracheit* or epiglottit*):ti,ab,kw
#18 MeSH descriptor: [Common Cold] this term only
#19 ((common near/2 cold*) or coryza or catarrh):ti,ab,kw
#20 MeSH descriptor: [Otitis Media] explode all trees
#21 otitis media:ti,ab,kw
#22 (AOM or OME):ti,ab,kw
#23 MeSH descriptor: [Cough] this term only
#24 cough*:ti,ab,kw
#25 MeSH descriptor: [Influenza, Human] this term only
#26 (influenza* or flu):ti,ab,kw
#27 MeSH descriptor: [Bronchitis] explode all trees
#28 MeSH descriptor: [Pneumonia] explode all trees
#29 (bronchit* or bronchiolit* or tracheobronchit* or pneumon* or bronchopneumon* or pleuropneumon*):ti,ab,kw
#30 MeSH descriptor: [Coronavirus] this term only
#31 MeSH descriptor: [Coronavirus Infections] explode all trees
#32 (coronavirus* or corona virus* or OC43 or NL63 or 229E or HKU1 or HCoV* or ncov* or COVID* or SARS-CoV* or sarscov* or Sars-coronavirus*):ti,ab,kw
#33 (2019-ncov or ncov19 or ncov-19 or 2019-novel CoV or SARS-CoV-2 or SARS-CoV-2 or SARS-CoV-2 or SARS-CoV-2 or Sars-coronavirus2 or Sars-coronavirus-2 or SARS-like coronavirus* or coronavirus-19 or COVID-19 or COVID-19 or COVID-19 or ((novel or new or nouveau) near/2 (CoV or nCoV or covid or coronavirus* or corona virus or Pandemi*2))):ti,ab,kw
#34 [9-#33]
#35 MeSH descriptor: [Meat] this term only
#36 MeSH descriptor: [Crab Meat] this term only
#37 MeSH descriptor: [Fish Meat] this term only
#38 MeSH descriptor: [Meat Juice] this term only
#39 MeSH descriptor: [Processed Meat] this term only
#40 MeSH descriptor: [Red Meat] explode all trees
#41 MeSH descriptor: [Smoked Meat] this term only
#42 MeSH descriptor: [White Meat] this term only
#43 MeSH descriptor: [Poultry Product] explode all trees
#44 MeSH descriptor: [Food] this term only
#45 MeSH descriptor: [Cooked Food] this term only
#46 MeSH descriptor: [Dietary Supplement] this term only
#47 MeSH descriptor: [Fermented Product] explode all trees
#48 MeSH descriptor: [Fortified Food] this term only
#49 MeSH descriptor: [Functional Food] this term only
#50 MeSH descriptor: [Health Food] this term only
#51 MeSH descriptor: [Instant Food] explode all trees
#52 MeSH descriptor: [Organic Food] this term only
#53 MeSH descriptor: [Poultry Product] explode all trees
#54 MeSH descriptor: [Preserved Food] explode all trees
#55 MeSH descriptor: [Processed Food] explode all trees
#56 MeSH descriptor: [Sea Food] explode all trees
#57 MeSH descriptor: [Soy Food] explode all trees
#58 MeSH descriptor: [Staple Food] explode all trees
#59 MeSH descriptor: [Whole Food] explode all trees
#60 MeSH descriptor: [Vegetable] explode all trees
#61 ((cold or hot or cook* or heat*) near/3 (soup* or broth* or stew* or chowder* or grav* or ramen*)):ti,ab,kw
#62 (((chicken or poultr*) near/2 (soup* or broth* or stew* or chowder*)) or (jewish near/2 penicillin) or bohbymycetin or bobamycin):ti,ab,kw
#63 (“canja de galinha” or ((sopa* or caldo) near/2 (ajo or pollo)) or bisque or chowder or gumbo or bouillabaisse or consomme or pot#age or minestrone or pho or gazpacho or borscht or hodgepodge or jambalaya or mulligatawny or sambar or “tom yum” or “kai tom” or vichyssoise or avgolemono or menudo or cioppino or goulash or “ash-e reshteh” or solyank or pozole or hominy or kharcho or gazpachuelo or “cullen skink” or gruel or brose):ti,ab,kw
#64 ((thai or indian or miso or japanese or soybean* or lentil* or “split pea” or french or root or potato* or leek* or mushroom* or cheese or garlic or cucumber* or beet* or greek or (egg near/2 lemon) or scotch or italian or american or seafood or clam* or oxtail or ham or vegetable* or cabbage or celery or pumpkin or squash* or onion* or yam* or hungarian or russian or spanish or fish or pork or persian or noodle* or pasta* or “egg drop” or chinese or louisiana or rice or meat* or georgian or beef or mexican or tortilla* or bone* or curr* or spic* or tofu or protein* or barley or dumpling* or dough* or sausage* or bacon or “matzo ball” or matzah or wonton* or pelmeni or turmeric or oat*) near/2 (soup* or broth* or stew* or chowder* or grav* or ramen*)):ti,ab,kw
#65 (“food as medicine” or (diet near/2 therap*) or (functional near/2 (food* or culinary or cook* or kitchen* or home* or domestic* or household) near/2 (remed* or medicine*))):ti,ab,kw
#66 {or #35-#65}
#67 #34 and #66

**MEDLINE Ovid search strategy**

1. respiratory tract infections/or nasopharyngitis/or exp sinusitis/or rhinitis/or sneezing/or pharyngitis/or tonsillitis/or exp laryngitis/or tracheitis/or epiglottitis/
2. (acute adj3 (chest or airway$ or bronchopulmonary or pulmonary or respirat$) adj3 disease$).tw.
3. ((respirat$ adj2 (failur$ or distress$ or infection$ or inflamm$ or disease$ or congest$ or syndrome$)) or (pain$ adj2 (respir$ or breath$))).tw.
4. (URTI or URI or LRTI or LRI or ARI or ARTI).tw.
5. (nasopharyngit$ or rhinopharyngit$ or sinusit$ or rhinit$ or rhinosinusit$ or nasosinusit$).tw.
6. (((runny or running or blocked or congest$ or discharg$ or stuffed or stuffy) adj2 (nose$ or nasal)) or rhinorrhea or rhinorrhoea or sneez$ or pharyngit$ or pharyngotonsillit$).tw.
7. (throat$ adj2 (sore or infect$ or inflam$)).tw.
8. (tonsillit$ or laryngit$ or croup$ or laryngotracheobronchit$ or pseudocroup$ or tracheit$ or rhinotracheit$ or epiglottit$).tw.
9. common cold/
10. ((common adj2 cold$) or coryza or catarrh).tw.
11. exp otitis media/
12. otitis media.tw.
13. (AOM or OME).tw.
14. cough/
15. cough$.tw.
16. influenza, human/
17. (influenza$ or flu).tw.
18. exp bronchitis/or exp pneumonia/
19. (bronchit$ or bronchiolit$ or tracheobronchit$ or pneumon$ or bronchopneumon$ or pleuropneumon$).tw.
20. exp coronavirus/
21. exp coronavirus infections/
22. (coronavirus$ or corona virus$ or OC43 or NL63 or 229E or HKU1 or HCoV$ or ncov$ or covid$ or sars-cov$ or sarscov$ or Sars-coronavirus$).tw.
23. (2019-ncov or ncov19 or ncov-19 or 2019-novel CoV or SARS-CoV-2 or SARS-CoV-2 or SARS-CoV-2 or SARS-CoV-2 or Sars-coronavirus2 or Sars-coronavirus-2 or SARS-like coronavirus$ or coronavirus-19 or COVID-19 or COVID-19 or COVID-19 or ((novel or new or nouveau) adj2 (CoV or nCoV or covid or coronavirus$ or corona virus or Pandemi$2))).tw.
24. or/1-23
25. meat/or crab meat/or fish meat/or meat juice/or processed meat/or exp red meat/or smoked meat/or exp white meat/
26. exp poultry product/
27. food/or cooked food/or dietary supplement/or exp fermented product/or fortified food/or functional food/or health food/or exp instant food/or organic food/or exp poultry product/or exp preserved food/or exp processed food/or exp sea food/or exp soy food/or staple food/or exp whole food/or exp vegetable/
28. ((cold or hot or cook$ or heat$) adj3 (soup$ or broth$ or stew$ or chowder$ or grav$ or ramen$)).tw.
29. (((chicken or poultr$) adj2 (soup$ or broth$ or stew$ or chowder$)) or (jewish adj2 penicillin) or bohbymycetin or bobamycin).tw.
30. (“canja de galinha” or ((sopa$ or caldo) adj2 (ajo or pollo)) or bisque or chowder or gumbo or bouillabaisse or consomme or pot#age or minestrone or pho or gazpacho or borscht or hodgepodge or jambalaya or mulligatawny or sambar or “tom yum” or “kai tom” or vichyssoise or avgolemono or menudo or cioppino or goulash or “ash-e reshteh” or solyank or pozole or hominy or kharcho or gazpachuelo or “cullen skink” or gruel or brose).tw.
31. ((thai or indian or miso or japanese or soybean$ or lentil$ or “split pea” or french or root or potato$ or leek$ or mushroom$ or cheese or garlic or cucumber$ or beet$ or greek or (egg adj2 lemon) or scotch or italian or american or seafood or clam$ or oxtail or ham or vegetable$ or cabbage or celery or pumpkin or squash$ or onion$ or yam$ or hungarian or russian or spanish or fish or pork or persian or noodle$ or pasta$ or “egg drop” or chinese or louisiana or rice or meat$ or georgian or beef or mexican or tortilla$ or bone$ or curr$ or spic$ or tofu or protein$ or barley or dumpling$ or dough$ or sausage$ or bacon or “matzo ball” or matzah or wonton$ or pelmeni or turmeric or oat$) adj2 (soup$ or broth$ or stew$ or chowder$ or grav$ or ramen$)).tw.
32. (“food as medicine” or (diet adj2 therap$) or (functional adj2 (food$ or culinary or cook$ or kitchen$ or home$ or domestic$ or household) adj2 (remed$ or medicine$))).tw.
33. or/25-32
34. randomized controlled trial.pt.
35. controlled clinical trial.pt.
36. randomized.ab.
37. placebo.ab.
38. clinical trials as topic.sh.
39. random$.ab.
40. or/34-39
41. 24 and 33 and 40

**Embase Ovid search strategy**

1. respiratory tract infection/or rhinopharyngitis/or pharyngitis/or rhinitis/or acute sinusitis/or sneezing/or tonsillitis/or laryngitis/or exp tracheitis/or acute epiglottitis/
2. (acute adj3 (chest or airway$ or bronchopulmonary or pulmonary or respirat$) adj3 disease$).tw.
3. ((respirat$ adj2 (failur$ or distress$ or infection$ or inflamm$ or disease$ or congest$ or syndrome$)) or (pain$ adj2 (respir$ or breath$))).tw.
4. (URTI or URI or LRTI or LRI or ARI or ARTI).tw.
5. (nasopharyngit$ or rhinopharyngit$ or sinusit$ or rhinit$ or rhinosinusit$ or nasosinusit$).tw.
6. (((runny or running or blocked or congest$ or discharg$ or stuffed or stuffy) adj2 (nose$ or nasal)) or rhinorrhea or rhinorrhoea or sneez$ or pharyngit$ or pharyngotonsillit$).tw.
7. (throat$ adj2 (sore or infect$ or inflam$)).tw.
8. (tonsillit$ or laryngit$ or croup$ or laryngotracheobronchit$ or pseudocroup$ or tracheit$ or rhinotracheit$ or epiglottit$).tw.
9. common cold/or nose infection/
10. ((common adj2 cold$) or coryza or catarrh).tw.
11. acute otitis media/
12. otitis media.tw.
13. (AOM or OME).tw.
14. coughing/or barking cough/or dry cough/or hacking cough/or irritative coughing/
15. cough$.tw.
16. influenza/
17. (influenza$ or flu).tw.
18. bronchitis/or pneumonia/
19. (bronchit$ or bronchiolit$ or tracheobronchit$ or pneumon$ or bronchopneumon$ or pleuropneumon$).tw.
20. coronaviridae/
21. coronavirus infection/or exp coronavirus disease 2019/or severe acute respiratory syndrome/
22. (coronavirus$ or corona virus$ or OC43 or NL63 or 229E or HKU1 or HCoV$ or ncov$ or covid$ or sars-cov$ or sarscov$ or Sars-coronavirus$).tw.
23. (2019-ncov or ncov19 or ncov-19 or 2019-novel CoV or SARS-CoV-2 or SARS-CoV-2 or SARS-CoV-2 or SARS-CoV-2 or Sars-coronavirus2 or Sars-coronavirus-2 or SARS-like coronavirus$ or coronavirus-19 or COVID-19 or COVID-19 or COVID-19 or ((novel or new or nouveau) adj2 (CoV or nCoV or covid or coronavirus$ or corona virus or Pandemi$2))).tw.
24. or/1-23
25. meat consumption/
26. food/or convenience food/or cooked food/or dietary supplement/or fast food/or fermented product/or exp instant food/or exp meat/or exp poultry product/or exp preserved food/or exp processed food/or exp sea food/or superfood/or exp vegetable/or exp whole food/
27. ((cold or hot or cook$ or heat$) adj3 (soup$ or broth$ or stew$ or chowder$ or grav$ or ramen$)).tw.
28. (((chicken or poultr$) adj2 (soup$ or broth$ or stew$ or chowder$)) or (jewish adj2 penicillin) or bohbymycetin or bobamycin).tw.
29. (“canja de galinha” or ((sopa$ or caldo) adj2 (ajo or pollo)) or bisque or chowder or gumbo or bouillabaisse or consomme or pot#age or minestrone or pho or gazpacho or borscht or hodgepodge or jambalaya or mulligatawny or sambar or “tom yum” or “kai tom” or vichyssoise or avgolemono or menudo or cioppino or goulash or “ash-e reshteh” or solyank or pozole or hominy or kharcho or gazpachuelo or “cullen skink” or gruel or brose).tw.
30. ((thai or indian or miso or japanese or soybean$ or lentil$ or “split pea” or french or root or potato$ or leek$ or mushroom$ or cheese or garlic or cucumber$ or beet$ or greek or (egg adj2 lemon) or scotch or italian or american or seafood or clam$ or oxtail or ham or vegetable$ or cabbage or celery or pumpkin or squash$ or onion$ or yam$ or hungarian or russian or spanish or fish or pork or persian or noodle$ or pasta$ or “egg drop” or chinese or louisiana or rice or meat$ or georgian or beef or mexican or tortilla$ or bone$ or curr$ or spic$ or tofu or protein$ or barley or dumpling$ or dough$ or sausage$ or bacon or “matzo ball” or matzah or wonton$ or pelmeni or turmeric or oat$) adj2 (soup$ or broth$ or stew$ or chowder$ or grav$ or ramen$)).tw.
31. (“food as medicine” or (diet adj2 therap$) or (functional adj2 (food$ or culinary or cook$ or kitchen$ or home$ or domestic$ or household) adj2 (remed$ or medicine$))).tw.
32. or/25-31
33. Randomized Controlled Trial/or “randomized controlled trial (topic)”/
34. Randomization/
35. Controlled clinical trial/or “controlled clinical trial (topic)”/
36. control group/or controlled study/
37. clinical trial/or “clinical trial (topic)”/or phase 1 clinical trial/or phase 2 clinical trial/or phase 3 clinical trial/or phase 4 clinical trial/
38. Crossover Procedure/
39. Double Blind Procedure/
40. Single Blind Procedure/or triple blind procedure/
41. placebo/or placebo effect/
42. (random$ or RCT or RCTs).tw.
43. (controlled adj5 (trial$ or stud$)).tw.
44. (clinical$ adj5 trial$).tw.
45. ((control or treatment or experiment$ or intervention) adj5 (group$ or subject$ or patient$)).tw. (quasi-random$ or quasi random$ or pseudo-random$ or pseudo random$).tw.
46. ((control or experiment$ or conservative) adj5 (treatment or therapy or procedure or manage$)).tw.
47. ((singl$ or doubl$ or tripl$ or trebl$) adj5 (blind$ or mask$)).tw.
48. (cross-over or cross over or crossover).tw.
49. (placebo$ or sham).tw.
50. trial.ti.
51. (assign$ or allocat$).tw.
52. controls.tw.
53. or/33-52
54. 24 and 32 and 53

**CINAHL EBSCO search strategy**

S1 (MH “Respiratory Tract Infections”) OR (MH “Common Cold”) OR (MH “Sinusitis+”) OR (MH “Severe Acute Respiratory Syndrome”) OR (MH “Rhinosinusitis”) OR (MH “Rhinitis”) OR (MH “Pneumonia+”) OR (MH “Pharyngitis”) OR (MH “Influenza”) OR (MH “Bronchitis, Acute”) OR (MH “Bronchiolitis”) OR (MH “Bronchitis”) OR (MH “Sneezing”)
S2 TI (acute n3 (chest or airway* or bronchopulmonary or pulmonary or respirat*) n3 disease*) OR AB (acute n3 (chest or airway* or bronchopulmonary or pulmonary or respirat*) n3 disease*)
S3 TI ((respirat* n2 (failur* or distress* or infection* or inflamm* or disease* or congest* or syndrome*)) or (pain* n2 (respir* or breath*))) OR AB ((respirat* n2 (failur* or distress* or infection* or inflamm* or disease* or congest* or syndrome*)) or (pain* n2 (respir* or breath*)))
S4 TI (URTI or URI or LRTI or LRI or ARI or ARTI) OR AB (URTI or URI or LRTI or LRI or ARI or ARTI)
S5 TI (nasopharyngit* or rhinopharyngit* or sinusit* or rhinit* or rhinosinusit* or nasosinusit*) OR AB (nasopharyngit* or rhinopharyngit* or sinusit* or rhinit* or rhinosinusit* or nasosinusit*)
S6 TI (((runny or running or blocked or congest* or discharg* or stuffed or stuffy) n2 (nose* or nasal)) or rhinorrhea or rhinorrhoea or sneez* or pharyngit* or pharyngotonsillit*) OR AB (((runny or running or blocked or congest* or discharg* or stuffed or stuffy) n2 (nose* or nasal)) or rhinorrhea or rhinorrhoea or sneez* or pharyngit* or pharyngotonsillit*)
S7 TI (throat* n2 (sore or infect* or inflam*)) OR AB (throat* n2 (sore or infect* or inflam*))
S8 TI (tonsillit* or laryngit* or croup* or laryngotracheobronchit* or pseudocroup* or tracheit* or rhinotracheit* or epiglottit*) OR AB (tonsillit* or laryngit* or croup* or laryngotracheobronchit* or pseudocroup* or tracheit* or rhinotracheit* or epiglottit*)
S9 TI ((common n2 cold*) or coryza or catarrh) OR AB ((common n2 cold*) or coryza or catarrh)
S10 (MH “Otitis Media+”)
S11 TI “otitis media” OR AB “otitis media”
S12 TI (AOM or OME)
S13 TI cough* OR AB cough*
S14 TI (bronchit* or bronchiolit* or tracheobronchit* or pneumon* or bronchopneumon* or pleuropneumon*) OR AB (bronchit* or bronchiolit* or tracheobronchit* or pneumon* or bronchopneumon* or pleuropneumon*)
S15 (MH “Coronavirus+”) OR (MH “COVID-19+”) OR (MH “Coronavirus Infections”)
S16 TI (influenza* or flu) OR AB (influenza* or flu)
S17 TI (coronavirus* or “corona virus*” or OC43 or NL63 or 229E or HKU1 or HCoV* or ncov* or covid* or sars-cov* or sarscov* or “Sars-coronavirus*”) OR AB (coronavirus* or “corona virus*” or OC43 or NL63 or 229E or HKU1 or HCoV* or ncov* or covid* or sars-cov* or sarscov* or “Sars-coronavirus*”)
S18 TI (2019-ncov or ncov19 or ncov-19 or 2019-novel CoV or SARS-CoV-2 or SARS-CoV-2 or SARS-CoV-2 or SARS-CoV-2 or Sars-coronavirus2 or Sars-coronavirus-2 or “SARS-like coronavirus*” or coronavirus-19 or COVID-19 or COVID-19 or “COVID-19” or ((novel or new or nouveau) n2 (CoV or nCoV or covid or coronavirus* or “corona virus” or Pandemi*))) OR AB (2019-ncov or ncov19 or ncov-19 or 2019-novel CoV or SARS-CoV-2 or SARS-CoV-2 or SARS-CoV-2 or SARS-CoV-2 or Sars-coronavirus2 or Sars-coronavirus-2 or “SARS-like coronavirus*” or coronavirus-19 or COVID-19 or COVID-19 or “COVID-19” or ((novel or new or nouveau) n2 (CoV or nCoV or covid or coronavirus* or “corona virus” or Pandemi*)))
S19 S1 OR S2 OR S3 OR S4 OR S5 OR S6 OR S7 OR S8 OR S9 OR 10 OR S11 OR S12 OR S13 OR S14 OR S15 OR S16 OR S17 OR S18
S20 (MH “Food”) OR (MH “Cereals+”) OR (MH “Dairy Products+”) OR (MH “Dietary Fats+”) OR (MH “Dietary Proteins+”) OR (MH “Dietary Supplements”) OR (MH “Eggs”) OR (MH “Food, Fortified”) OR (MH “Foods, Fermented+”) OR (MH “Health Food+”) OR (MH “Herbs, Seasoning”) OR (MH “Meat+”) OR (MH “Mushroom, Edible”) OR (MH “Nutrients+”) OR (MH “Nuts+”) OR (MH “Vegetables+”) OR (MH “Soy Foods+”)
S21 TI ((cold or hot or cook* or heat*) n3 (soup* or broth* or stew* or chowder* or grav* or ramen*)) OR AB ((cold or hot or cook* or heat*) n3 (soup* or broth* or stew* or chowder* or grav* or ramen*))
S22 TI (((chicken or poultr*) n2 (soup* or broth* or stew* or chowder*)) or (jewish n2 penicillin) or bohbymycetin or bobamycin) OR AB (((chicken or poultr*) n2 (soup* or broth* or stew* or chowder*)) or (jewish n2 penicillin) or bohbymycetin or bobamycin)
S23 TI (“canja de galinha” or ((sopa* or caldo) n2 (ajo or pollo)) or bisque or chowder or gumbo or bouillabaisse or consomme or pot#age or minestrone or pho or gazpacho or borscht or hodgepodge or jambalaya or mulligatawny or sambar or “tom yum” or “kai tom” or vichyssoise or avgolemono or menudo or cioppino or goulash or “ash-e reshteh” or solyank or pozole or hominy or kharcho or gazpachuelo or “cullen skink” or gruel or brose) OR AB (“canja de galinha” or ((sopa* or caldo) n2 (ajo or pollo)) or bisque or chowder or gumbo or bouillabaisse or consomme or pot#age or minestrone or pho or gazpacho or borscht or hodgepodge or jambalaya or mulligatawny or sambar or “tom yum” or “kai tom” or vichyssoise or avgolemono or menudo or cioppino or goulash or “ash-e reshteh” or solyank or pozole or hominy or kharcho or gazpachuelo or “cullen skink” or gruel or brose)
S24 TI ((thai or indian or miso or japanese or soybean* or lentil* or “split pea” or french or root or potato* or leek* or mushroom* or cheese or garlic or cucumber* or beet* or greek or (egg n2 lemon) or scotch or italian or american or seafood or clam* or oxtail or ham or vegetable* or cabbage or celery or pumpkin or squash* or onion* or yam* or hungarian or russian or spanish or fish or pork or persian or noodle* or pasta* or “egg drop” or chinese or louisiana or rice or meat* or georgian or beef or mexican or tortilla* or bone* or curr* or spic* or tofu or protein* or barley or dumpling* or dough* or sausage* or bacon or “matzo ball” or matzah or wonton* or pelmeni or turmeric or oat*) n2 (soup* or broth* or stew* or chowder* or grav* or ramen*)) OR AB ((thai or indian or miso or japanese or soybean* or lentil* or “split pea” or french or root or potato* or leek* or mushroom* or cheese or garlic or cucumber* or beet* or greek or (egg n2 lemon) or scotch or italian or american or seafood or clam* or oxtail or ham or vegetable* or cabbage or celery or pumpkin or squash* or onion* or yam* or hungarian or russian or spanish or fish or pork or persian or noodle* or pasta* or “egg drop” or chinese or louisiana or rice or meat* or georgian or beef or mexican or tortilla* or bone* or curr* or spic* or tofu or protein* or barley or dumpling* or dough* or sausage* or bacon or “matzo ball” or matzah or wonton* or pelmeni or turmeric or oat*) n2 (soup* or broth* or stew* or chowder* or grav* or ramen*))
S25 TI (“food as medicine” or (diet n2 therap*) or (functional n2 (food* or culinary or cook* or kitchen* or home* or domestic* or household) n2 (remed* or medicine*))) OR AB (“food as medicine” or (diet n2 therap*) or (functional n2 (food* or culinary or cook* or kitchen* or home* or domestic* or household) n2 (remed* or medicine*)))
S26 S20 OR S21 OR S22 OR S23 OR S24 OR S25
S27 S26 OR S19

**Web of Science (results from Science Citation Index Expanded (SCI-EXPANDED), Conference Proceedings Citation Index-Science (CPCI-S)) search strategy**

#1 TS = (acute near/3 (chest or airway* or bronchopulmonary or pulmonary or respirat*) near/3 disease*)
#2 TS = ((respirat* near/2 (failur* or distress* or infection* or inflamm* or disease* or congest* or syndrome*)) or (pain* near/2 (respir* or breath*)))
#3 TS = (URTI or URI or LRTI or LRI or ARI or ARTI)
#4 TS = (nasopharyngit* or rhinopharyngit* or sinusit* or rhinit* or rhinosinusit* or nasosinusit*)
#5 TS = (((runny or running or blocked or congest* or discharg* or stuffed or stuffy) near/2 (nose* or nasal)) or rhinorrhea or rhinorrhoea or sneez* or pharyngit* or pharyngotonsillit*)
#6 TS = (throat* near/2 (sore or infect* or inflam*))
#7 TS = (tonsillit* or laryngit* or croup* or laryngotracheobronchit* or pseudocroup* or tracheit* or rhinotracheit* or epiglottit*)
#8 TS = ((common near/2 cold*) or coryza or catarrh)
#9 TS = “otitis media”
#10 TS = (AOM or OME)
#11 TS = cough*
#12 TS = (influenza* or flu)
#13 TS = (bronchit* or bronchiolit* or tracheobronchit* or pneumon* or bronchopneumon* or pleuropneumon*)
#14 TS = (coronavirus* or “corona virus*” or OC43 or NL63 or 229E or HKU1 or HCoV* or ncov* or covid* or sars-cov* or sarscov* or “Sars-coronavirus*”)
#15 TS = (2019-ncov or ncov19 or ncov-19 or 2019-novel CoV or SARS-CoV-2 or SARS-CoV-2 or SARS-CoV-2 or SARS-CoV-2 or Sars-coronavirus2 or Sars-coronavirus-2 or “SARS-like coronavirus*” or coronavirus-19 or COVID-19 or COVID-19 or “COVID-19” or
#16 ((novel or new or nouveau) near/2 (CoV or nCoV or covid or coronavirus* or “corona virus” or Pandemi*)))
#17 #1 OR #2 OR #3 OR #4 OR #5 OR #6 OR #7 OR #8 OR #9 OR 10 OR #11 OR #12 OR #13 OR #14 OR #15 OR #16
#18 TS = ((cold or hot or cook* or heat*) near/3 (soup* or broth* or stew* or chowder* or grav* or ramen*))
#19 TS = (((chicken or poultr*) near/2 (soup* or broth* or stew* or chowder*)) or (jewish near/2 penicillin) or bohbymycetin or bobamycin)
#20 TS = (“canja de galinha” or ((sopa* or caldo) near/2 (ajo or pollo)) or bisque or chowder or gumbo or bouillabaisse or consomme or pot?age or minestrone or pho or gazpacho or borscht or hodgepodge or jambalaya or mulligatawny or sambar or “tom yum” or “kai tom” or vichyssoise or avgolemono or menudo or cioppino or goulash or “ash-e reshteh” or solyank or pozole or hominy or kharcho or gazpachuelo or “cullen skink” or gruel or brose)
#21 TS = ((thai or indian or miso or japanese or soybean* or lentil* or “split pea” or french or root or potato* or leek* or mushroom* or cheese or garlic or cucumber* or beet* or greek or (egg near/2 lemon) or scotch or italian or american or seafood or clam* or oxtail or ham or vegetable* or cabbage or celery or pumpkin or squash* or onion* or yam* or hungarian or russian or spanish or fish or pork or persian or noodle* or pasta* or “egg drop” or chinese or louisiana or rice or meat* or georgian or beef or mexican or tortilla* or bone* or curr* or spic* or tofu or protein* or barley or dumpling* or dough* or sausage* or bacon or “matzo ball” or matzah or wonton* or pelmeni or turmeric or oat*) near/2 (soup* or broth* or stew* or chowder* or grav* or ramen*))
#22 TS = (“food as medicine” or (diet near/2 therap*) or (functional near/2 (food* or culinary or cook* or kitchen* or home* or domestic* or household) near/2 (remed* or medicine*)))
#23 #18 or #19 or #20 or #21 or #22
#24 TS = trial
#25 TS = random*
#26 #24 or #25
#27 #17 and #23 and #26

**SCOPUS search strategy**

1. TITLE-ABS-KEY ((acute PRE/3 (chest OR airway* OR bronchopulmonary OR pulmonary OR respirat*) PRE/3 disease*))
2. TITLE-ABS-KEY (((respirat* PRE/2 (failur* OR distress* OR infection* OR inflamm* OR disease* OR congest* OR syndrome*)) OR (pain* W/2 (respir* OR breath*))))
3. TITLE-ABS-KEY ((urti OR uri OR lrti OR lri OR ari OR arti))
4. TITLE-ABS-KEY ((nasopharyngit* OR rhinopharyngit* OR sinusit* OR rhinit* OR rhinosinusit* OR nasosinusit*))
5. TITLE-ABS-KEY ((((runny OR running OR blocked OR congest* OR discharg* OR stuffed OR stuffy) W/2 (nose* OR nasal)) OR rhinorrhea OR rhinorrhoea OR sneez* OR pharyngit* OR pharyngotonsillit*))
6. TITLE-ABS-KEY ((throat* W/2 (sore or infect* or inflam*)))
7. TITLE-ABS-KEY ((tonsillit* or laryngit* or croup* or laryngotracheobronchit* or pseudocroup* or tracheit* or rhinotracheit* or epiglottit*))
8. TITLE-ABS-KEY (((common PRE/2 cold*) OR coryza OR catarrh))
9. TITLE-ABS-KEY (otitis media)
10. TITLE-ABS-KEY ((AOM or OME))
11. TITLE-ABS-KEY (cough*)
12. TITLE-ABS-KEY ((influenza* or flu))
13. TITLE-ABS-KEY ((bronchit* or bronchiolit* or tracheobronchit* or pneumon* or bronchopneumon* or pleuropneumon*))
14. TITLE-ABS-KEY ((coronavirus* or corona virus* or OC43 or NL63 or 229E or HKU1 or HCoV* or ncov* or covid* or sars-cov* or sarscov* or Sars-coronavirus*))
15. TITLE-ABS-KEY (2019-ncov OR ncov19 OR ncov-19 OR “2019-novel cov” OR SARS-CoV-2 OR SARS-CoV-2 OR SARS-CoV-2 OR SARS-CoV-2 OR sars-coronavirus2 OR sars-coronavirus-2 OR (sars-like AND coronavirus*) OR coronavirus-19 OR COVID-19 OR COVID-19 OR COVID-19)
16. TITLE-ABS-KEY (((novel OR new OR nouveau) W/2 (cov OR ncov OR covid OR coronavirus* OR “corona virus” OR pandemi*)))
17. 1 or 2 or 3 or 4 or 5 or 6 or 7 or 8 or 9 or 10 or 11 or 12 or 13 or 14 or 15 or 16
18. TITLE-ABS-KEY (((cold OR hot OR cook* OR heat*) W/3 (soup* OR broth* OR stew* OR chowder* OR grav* OR ramen*)))
19. TITLE-ABS-KEY (((chicken or poultr*) PRE/2 (soup* or broth* or stew* or chowder*)) or (jewish PRE/2 penicillin) or bohbymycetin or bobamycin)
20. TITLE-ABS-KEY ((“canja de galinha” OR ((sopa* OR caldo) W/2 (ajo OR pollo)) OR bisque OR chowder OR gumbo OR bouillabaisse OR consomme OR pottage OR minestrone OR pho OR gazpacho OR borscht OR hodgepodge OR jambalaya OR mulligatawny OR sambar OR “tom yum” OR “kai tom” OR vichyssoise OR avgolemono OR menudo OR cioppino OR goulash OR “ash-e reshteh” OR solyank OR pozole OR hominy OR kharcho OR gazpachuelo OR “cullen skink” OR gruel OR brose))
21. TITLE-ABS-KEY (((thai OR indian OR miso OR japanese OR soybean* OR lentil* OR “split pea” OR french OR root OR potato* OR leek* OR mushroom* OR cheese OR garlic OR cucumber* OR beet* OR greek OR (egg W/2 lemon) OR scotch OR italian OR american OR seafood OR clam* OR oxtail OR ham OR vegetable* OR cabbage OR celery OR pumpkin OR squash* OR onion* OR yam* OR hungarian OR russian OR spanish OR fish OR pork OR persian OR noodle* OR pasta* OR “egg drop” OR chinese OR louisiana OR rice OR meat* OR georgian OR beef OR mexican OR tortilla* OR bone* OR curr* OR spic* OR tofu OR protein* OR barley OR dumpling* OR dough* OR sausage* OR bacon OR “matzo ball” OR matzah OR wonton* OR pelmeni OR turmeric OR oat*) W/2 (soup* OR broth* OR stew* OR chowder* OR grav* OR ramen*)))
22. TITLE-ABS-KEY ((“food as medicine” OR (diet PRE/2 therap*) OR (functional PRE/2 (food* OR culinary OR cook* OR kitchen* OR home* OR domestic* OR household OR remed* OR medicine*))))
23. 18 or 19 or 20 or 21 or 22
24. TITLE-ABS-KEY (trial OR random*)
25. 17 and 23 and 24

**US National Institutes of Health Ongoing Trials Register ClinicalTrials.gov (www.clinicaltrials.gov)**

Meat OR Food OR Soup OR Diet, Healthy|Interventional Studies|Airways OR Respiratory OR Common Cold OR Pneumonia OR Throat

**World Health Organization International Clinical Trials Registry Platform (apps.who.int/trialsearch)**

Intervention: meat or food or soup or diet, healthy

Condition: airways or respiratory or common cold or pneumonia or throat

Phases: ALL
